# Inflammation promotes adipocyte lipolysis *via* IRE1 kinase

**DOI:** 10.1016/j.jbc.2021.100440

**Published:** 2021-02-19

**Authors:** Kevin P. Foley, Yong Chen, Nicole G. Barra, Mark Heal, Kieran Kwok, Akhilesh K. Tamrakar, Wendy Chi, Brittany M. Duggan, Brandyn D. Henriksbo, Yong Liu, Jonathan D. Schertzer

**Affiliations:** 1 Department of Biochemistry and Biomedical Sciences, Farncombe Family Digestive Health Research Institute and Centre for Metabolism, Obesity, and Diabetes Research McMaster University, Hamilton, Ontario, Canada; 2 Hubei Key Laboratory of Cell Homeostasis, College of Life Sciences, The Institute for Advanced Studies, Wuhan University, Wuhan, China; 3 Division of Biochemistry, CSIR-Central Drug Research Institute, Lucknow, India

**Keywords:** obesity, lipid, ER stress, endocrinology, metabolic syndrome, inflammation, adipocyte, lipolysis, cytokine, immunometabolism, 8-Br-cAMP, 8-bromoadenosine 3′,5′-cyclic monophosphate, 4-PBA, 4-phenylbutyric acid, ABL, Abelson murine leukemia viral oncogene homolog, AKO, adipose knockout, ATF6, activating transcription factor 6, ATGL, adipose triglyceride lipase, BMDM, bone-marrow-derived macrophage, CHOP, C/EBP homologous protein, ER, endoplasmic reticulum, ERK, extracellular signal-regulated kinase, FK565, heptanoyl-γ-D-glutamyl-L-meso-diamino-pimelyl-D-alanine, Grp78/BiP, binding immunoglobulin protein or heat shock 70 kDa protein 5, HEK, human embryonic kidney, HSL, hormone sensitive lipase, Il(6), interleukin, IRE1, inositol-requiring enzyme 1, KirA6, IRE1α kinase inhibiting RNase attenuator 6, NF-κB, nuclear factor kappa-light-chain-enhancer of activated B cells, LPS, lipopolysaccharide, NOD, nucleotide-binding oligomerization domain-containing protein, PERK, protein kinase RNA-like ER kinase, PGN, peptidoglycan, PKA, protein kinase A, PVDF, polyvinylidene difluoride, Akt, protein kinase B, RIPK2, receptor interacting serine/threonine kinase 2, SEAP, secreted alkaline phosphatase, SVF, stromal vascular fraction, TLR4, toll-like receptor 4, TKI, tyrosine kinase inhibitor, TNF, tumor necrosis factor, TUDCA, tauroursodeoxycholic acid, UPR, unfolded protein response, WAT, white adipose tissue, WT, wild type, XBP1, X-box binding protein 1

## Abstract

Obesity associates with inflammation, insulin resistance, and higher blood lipids. It is unclear if immune responses facilitate lipid breakdown and release from adipocytes *via* lipolysis in a separate way from hormones or adrenergic signals. We found that an ancient component of ER stress, inositol-requiring protein 1 (IRE1), discriminates inflammation-induced adipocyte lipolysis versus lipolysis from adrenergic or hormonal stimuli. Our data show that inhibiting IRE1 kinase activity was sufficient to block adipocyte-autonomous lipolysis from multiple inflammatory ligands, including bacterial components, certain cytokines, and thapsigargin-induced ER stress. IRE1-mediated lipolysis was specific for inflammatory triggers since IRE1 kinase activity was dispensable for isoproterenol and cAMP-induced lipolysis in adipocytes and mouse adipose tissue. IRE1 RNase activity was not associated with inflammation-induced adipocyte lipolysis. Inhibiting IRE1 kinase activity blocked NF-κB activation, interleukin-6 secretion, and adipocyte-autonomous lipolysis from inflammatory ligands. Inflammation-induced lipolysis mediated by IRE1 occurred independently from changes in insulin signaling in adipocytes, suggesting that inflammation can promote IRE1-mediated lipolysis independent of adipocyte insulin resistance. We found no role for canonical unfolded protein responses or ABL kinases in linking ER stress to IRE1-mediated lipolysis. Adiponectin-Cre-mediated IRE1 knockout in mice showed that adipocyte IRE1 was required for inflammatory ligand-induced lipolysis in adipose tissue explants and that adipocyte IRE1 was required for approximately half of the increase in blood triglycerides after a bacterial endotoxin-mediated inflammatory stimulus *in vivo*. Together, our results show that IRE1 propagates an inflammation-specific lipolytic program independent from hormonal or adrenergic regulation. Targeting IRE1 kinase activity may benefit metabolic syndrome and inflammatory lipid disorders.

Obesity and metabolic diseases are pervasive (1, 2). Some consequences of obesity are underpinned by compartmentalized immune responses in tissues that participate in metabolic regulation (3). This “metaflammation” can manifest from autocrine responses within metabolic cell types that regulate glucose and lipid metabolism (4). Paracrine communication between immune and metabolic cells can also impair local and systemic endocrine and metabolic responses (5, 6, 7). For example, inflammation of metabolic tissues can manifest as insulin resistance (8). However, the cellular nodes that dictate metabolic defects linked to inflammation-specific responses are ill-defined.

Adipocyte expansion during obesity coincides with increased adipose tissue inflammation, including aberrant cytokine production from both adipocytes and adipose-resident immune cells (9). Obesity, metabolic stress, and inflammation in the adipose tissue environment are also associated with adipocyte insulin resistance, including reduced ability of insulin to inhibit lipolysis (9, 10). There is a bidirectional link between lipolysis and adipocyte inflammation. Inflammatory mediators, such as tumor necrosis factor (TNF) and interleukin (Il)6, can increase adipocyte lipolysis (11, 12). Elevated levels of saturated free fatty acids (13, 14) and even lipolysis itself can promote cellular and tissue inflammation (15). Further, components of the intestinal microbiota can alter adipocyte lipolysis. Gut-derived bacterial components can penetrate into adipose tissue compartments (16). Bacterial components such as lipopolysaccharide (LPS) and peptidoglycan (PGN) are inflammatory ligands and stimuli for increased lipolysis (17, 18). Systemic levels of LPS and PGN are increased during obesity, positioning these bacterial factors as a connection between inflammation, adipose tissue metabolism, and insulin resistance (19, 20, 21, 22, 23). In fact, PGN acting on nucleotide-binding oligomerization domain-containing protein 1 (NOD1) and LPS acting on Toll-like receptor 4 (TLR4) within adipocytes can promote inflammation and lipolysis (17, 18, 24).

Excessive lipolysis can result in the accumulation of ectopic lipids in the liver and skeletal muscles, which can subsequently contribute to systemic insulin resistance (25). Increased lipolysis during obesity is often formulated in terms of adipocyte insulin resistance. However, inflammation-induced lipolysis can occur independently of changes in insulin action (17, 18, 26). More importantly, it is unclear if certain cellular triggers are specific to inflammation-induced lipolysis compared with adrenergic control of lipolysis, which governs adipocyte metabolism and lipid flux *via* catecholamines and other hormones. We hypothesized that only inflammation engaged ancient endoplasmic reticulum (ER) stress responses to provoke lipolysis, independently of adrenergic or hormonal influence on lipolysis, including insulin resistance.

Seminal work discovered how components of the ER stress response link inflammation to defects in glucose and lipid metabolism (27, 28). For example, nitrosylation of inositol-requiring protein 1 (IRE1) can compromise the unfolded protein response (UPR) and promote insulin resistance in obese mice (29). ER stress components include IRE1, activating transcription factor 6 (ATF6), and protein kinase RNA-like ER kinase (PERK). Much of the work on metabolic disease and ER stress has focussed on the UPR aspects of these components. For example, IRE1 can integrate stress signals from changes in cellular metabolism and nutrient levels coincident with overload of misfolded proteins (30). We questioned whether ER stress could propagate stress from inflammatory cues into a lipolytic program in adipocytes and whether the UPR or other kinases were involved. There is evidence that ER stress can promote adipocyte lipolysis coincident with elevated cAMP and activation of protein kinase A (PKA) (31). Further, ER stress can engage extracellular signal-regulated kinase (ERK)2 to promote hormone sensitive lipase (HSL)-mediated lipolysis (31). However, it is not known if inflammation uses a specific component of ER stress or a specific kinase to propagate lipolysis. Inflammatory triggers of lipolysis may engage an ER stress component, since bacterial LPS and PGN engage ERK2 and other kinases to promote lipolysis in adipocytes (17, 18). Recent evidence has also shown that ERK2 promoted serine 247 phosphorylation and activation of the Beta3 adrenoreceptor and increased adipocyte lipolysis during obesity (32). This seminal work showed that adrenergic stimuli engaged an ERK2-mediated stress response to promote lipolysis (32). Thus, kinases can communicate with adrenergic responses to promote lipolysis. However, it is not clear if inflammatory triggers of lipolysis, such as bacteria or cytokines, require a specific kinase linked to the ER stress response to promote lipolysis. This is relevant to metabolic disease since there are links between ER stress, inflammation, and increased lipolysis during obesity. We sought to define if a kinase inherent to ER stress components discriminates immune versus hormonal regulation of lipolysis. It is known that knockdown of IRE1 mitigates fasting-induced lipolysis in *C. elegans* (33). Here, we report that IRE1 was necessary for inflammation-induced lipolysis in adipocytes. We then questioned whether lipolysis was linked to the kinase activity of IRE1 or the canonical ER stress response regulated by IRE1. IRE1 is well known to promote endoribonuclease (RNase) cleavage of X-box binding protein 1 (XBP1) mRNA resulting in spliced XBP1 in response to luminal overload of unfolded proteins (34). Spliced XBP1 tips the balance away from lipogenesis toward lipolysis by augmenting macrolipophagy in the liver (35). Little is known about IRE1 in adipose tissue lipolysis or a potential independent role for IRE1 kinase activity in lipid metabolism. There is a growing interest in the kinase activity of IRE1 in linking inflammation and metabolism and as a therapeutic target (30, 36). The kinase and RNase activities of IRE1 can have opposing roles in ER stress responses to specific stressors such as viral replication (37). We tested if IRE1 propagated inflammation-rated metabolic dysfunction in adipocytes and determined if the UPR-RNase activity or kinase activity of IRE1 transmitted inflammation or adrenergic-specific signals into increased lipolysis in adipose tissue. We found that IRE1 kinase activity regulates inflammation-specific lipolysis without engaging canonical ER stress signaling involving IRE1 RNase. Inflammatory triggers of adipocyte lipolysis engage IRE1 kinase in a response that is separate from adrenergic or hormonal triggers of lipolysis.

## Results

### IRE1 kinase activity mediates inflammation-induced lipolysis.

**Figure 1:**
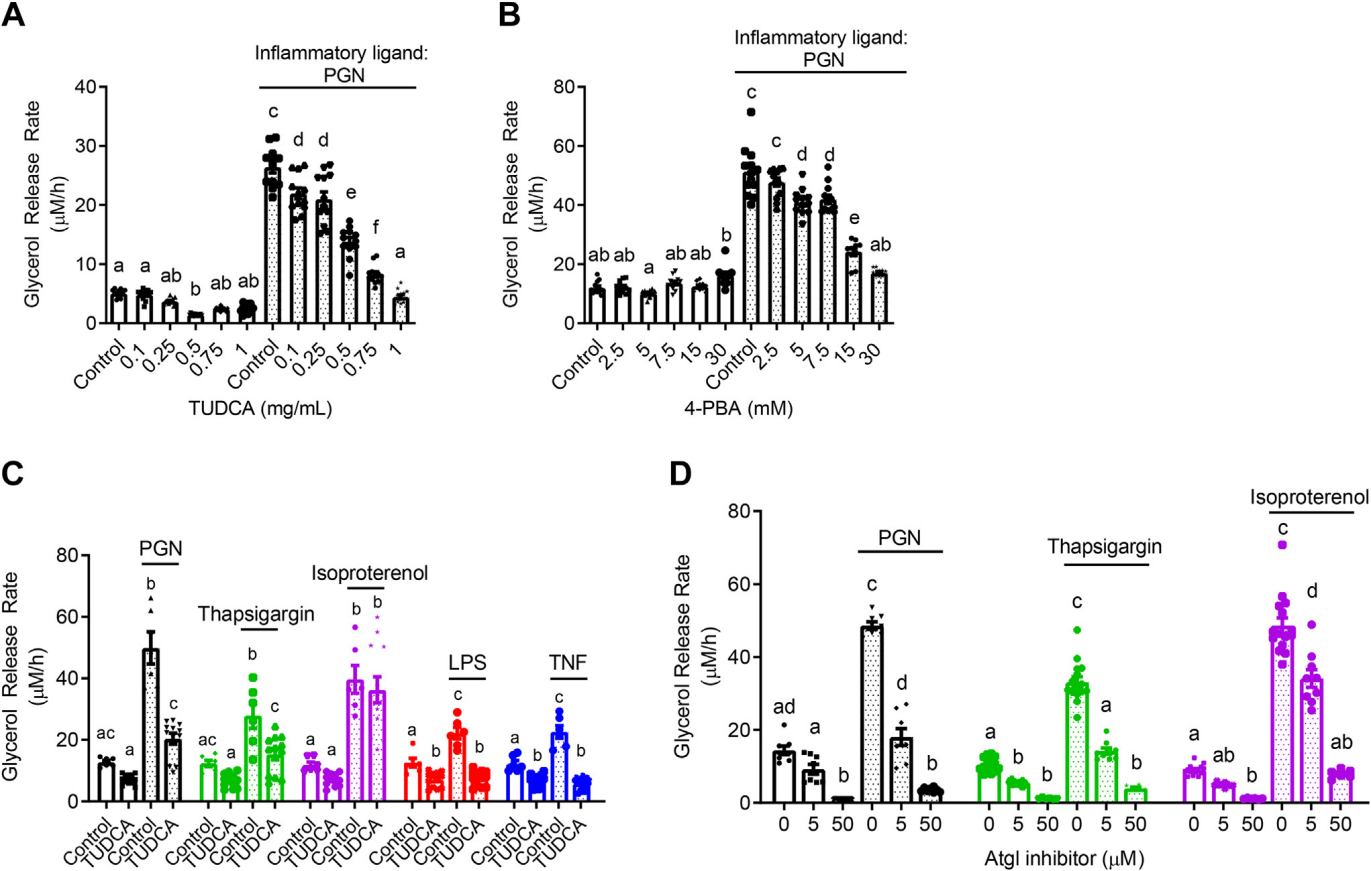
**ER stress mediates inflammation-induced lipolysis.***A* and *B*, glycerol release rates (μM/h) in 3T3-L1 adipocytes treated with increasing concentrations of the chemical chaperones TUDCA (*A*; N = 12) or 4-PBA (*B*; N = 10–12) in the absence (*white bars*) or presence (*dotted bars*) of the inflammatory ligand peptidoglycan (PGN) using 10 μg/ml FK565 *C*) glycerol release rates in 3T3-L1 adipocytes measured in the presence or absence of 1 mg/ml TUDCA, treated with vehicle (mock treatment), 10 μg/ml PGN, 1 μM thapsigargin, 2 μM isoproterenol, 500 ng/ml LPS, or 10 ng/ml TNF (N = 6–12). *D*, glycerol release rates in 3T3-L1 adipocytes treated with increasing concentrations of the ATGL inhibitor Atglistatin in the absence or presence of 10 μg/ml PGN, 1 μM thapsigargin, or 2 μM isoproterenol (N = 6–15). Values are mean ± SEM. Statistical significance was measured as *p* < 0.05 using two-way ANOVA. Post hoc analysis was performed using Tukey’s multiple comparisons test. Conditions with different letters (a, b) denote a statistical difference compared with all other conditions without the same letter.

To determine if ER stress propagated adipocyte lipolysis from an inflammatory stimulus, differentiated 3T3-L1 adipocytes were treated with or without 10 μg/ml NOD1 ligand (FK565) and increasing concentrations of the chemical chaperons TUDCA or 4-PBA (Fig. 1, *A* and *B*). We have previously determined the dose–response relationship of FK565 on lipolysis (18). FK565, a synthetic mimetic of bacterial cell wall PGN typical of Gram-negative bacteria, stimulated an approximately fivefold increase in glycerol release rate, which was dose-dependently inhibited by TUDCA and 4-PBA (Fig. 1, *A* and *B*). These results indicate that some aspect of ER stress participates in bacterial cell wall stimulated adipocyte lipolysis. We next examined if ER stress was required for induction of lipolysis with multiple inflammatory ligands compared with lipolysis caused by the adrenergic stimulus, isoproterenol. We used the inflammatory stimuli PGN (FK565 10 μg/L = NOD1 activation), LPS (500 ng/ml = TLR4 activation), TNF (10 ng/ml = paracrine cytokine), or thapsigargin (1 μM = ER stress activation), all of which increased the glycerol release rate in 3T3-L1 adipocytes. The increased lipolysis from all of these stimuli was blocked by 1 mg/ml TUDCA (Fig. 1C). However, glycerol release induced by the β-adrenergic agonist isoproterenol (2 μM) was not altered by TUDCA (Fig. 1*C*). These results suggest that ER stress may discriminate lipolysis from inflammation compared with catecholamines. We next tested the sensitivity of FK565, thapsigargin, and isoproterenol stimulated lipolysis to ATGL inhibition using Atglistatin in 3T3-L1 adipocytes. Inhibition of ATGL with Atglistatin blocked lipolysis induced by each of these ligands, confirming that glycerol release marked canonical activation of an ATGL-sensitive lipolytic program in adipocytes (Fig. 1*D*).

**Figure 2:**
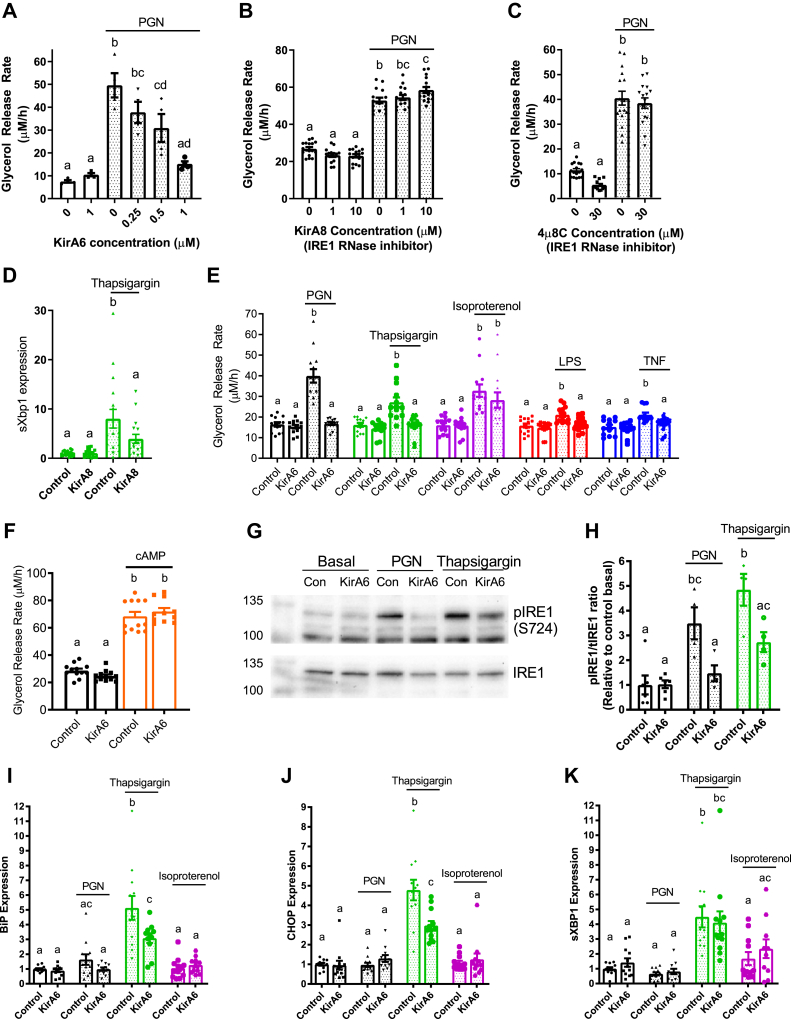
**Inflammatory ligands stimulate lipolysis *via* IRE1 kinase not RNase activity.***A–C*, glycerol release rates (μM/h) in 3T3-L1 adipocytes treated with increasing concentrations of the IRE1 inhibitor KirA6 (*A*; N = 4), the IRE1 RNase inhibitor KirA8 (*B*; N = 16), or the IRE1 RNase inhibitor 4μ8C (*C*; N = 12–16). *D*, expression of spliced Xbp1 in 3T3-L1 adipocytes treated with 1 μM Thapsigargin for 3 h with and without 10 μM KirA8. *E*, glycerol release rates in differentiated 3T3-L1 adipocytes treated with or without 1 μM KirA6 (IRE1 inhibitor) and one of vehicle (mock treatment), 10 μg/ml FK565 (PGN), 1 μM thapsigargin (ER stress activator), 2 μM isoproterenol (β-adrenergic agonist), 500 ng/ml LPS (TLR4 activator), or 10 ng/ml TNF, as indicated (N = 12–14). *F*, glycerol release rates in 3T3-L1 adipocytes treated with 1 μM KirA6 and 0.5 mM 8-Br-cAMP (N = 10–12). *G*, phosphorylation of IRE1 was measured in 3T3-L1 adipocytes after stimulation with vehicle, PGN (10 μg/ml), or thapsigargin (1 μM) for 3 h, with or without 1 μM KirA6 (N = 4–6). Blots were stripped and reprobed for total IRE1. *H*, quantification of western blots for the ratio between phosphorylated IRE1 and total IRE1, expressed relative to the basal control sample. *I–K*, RNA isolated from 3T3-L1 adipocytes was treated for 3 h with vehicle, FK565 (PGN, 10 μg/ml), thapsigargin (1 μM), or isoproterenol (2 μM) in the absence or presence of KirA6 (1 μM). Expression of BiP (*I*), CHOP (*J*), and spliced Xbp1(K) (N = 12). Values are mean ± SEM. Statistical significance was measured as *p* < 0.05 using two-way ANOVA. Post hoc analysis was performed using Tukey’s multiple comparisons test. Conditions with different letters (a, b) denote a statistical difference compared with all other conditions without the same letter.

IRE1 has been shown to link ER stress to NOD1/2-mediated immune responses (38, 39). We examined if IRE1 was the component of ER stress required for lipolysis stimulated by multiple inflammatory factors compared with adrenergic/hormonal pathways. Glycerol release was measured in 3T3-L1 cells treated with KirA6, an IRE1 kinase inhibitor (which also attenuates RNase activity), or one of two IRE1 RNase inhibitors, KirA8 and 4μ8C (Fig. 2, *A–C*). KirA6 dose-dependently lowered PGN-mediated lipolysis, where 1 μM KirA6 blocked an increase in lipolysis (Fig. 2*A*). KirA8 and 4μ8C did not lower glycerol release (Fig. 2, *B* and *C*). We also confirmed that 10 μM KirA8 blocked the RNase activity of IRE1 evinced by lower levels of spliced XBP1 in adipocytes treated with thapsigargin (Fig. 2*D*). These data suggest that IRE1 kinase activity (but not RNase activity) propagates inflammation-induced adipocyte lipolysis from a bacterial cell wall component. To test the role of IRE1 in mediating lipolysis induced by other inflammatory ligands, differentiated 3T3-L1 cells were treated with or without the IRE1 inhibitor KirA6 (1 μM) for 1 h before treatment with the inflammatory stimuli PGN, LPS, TNF, or thapsigargin. KirA6 completely blocked lipolysis stimulated by each of these inflammatory stimuli (Fig. 2*E*). In contrast, KirA6 did not inhibit lipolysis stimulated by the hormonal stimulus isoproterenol (2 μM) (Fig. 2*E*). We also confirmed that KirA6 did not change 8-Br-cAMP-induced lipolysis in adipocytes (Fig. 2*F*). These data demonstrate that IRE1 kinase activity is required for inflammatory, but not adrenergic-cAMP-stimulated, lipolysis in adipocytes.

We next examined IRE1 phosphorylation at Serine-724 (to biomark kinase activity) and transcript levels of key UPR intermediates (that are regulated by RNase activity). Treatment of 3T3-L1 adipocytes with PGN or thapsigargin for 3 h increased the levels of phosphorylated IRE1 (S724) relative to total levels of IRE1 (tIRE1) in 3T3 adipocytes, an effect that was inhibited by 1 μM KirA6 (Fig. 2, *G* and *H*). After this same 3-h treatment, thapsigargin, but not PGN or isoproterenol, increased the expression of BiP, CHOP, and sXBP1 (Fig. 2, *I–K*). These data show that neither bacterial ligand (*i.e.*, PGN) nor adrenergic stimulus (isoproterenol) increases ER stress markers typical of the UPR in adipocytes. Further, the increase in expression of BiP and CHOP induced by thapsigargin was sensitive to KirA6, whereas higher levels of spliced XBP1 (sXBP1), a key target of IRE1 RNase activity, were not affected by KirA6. These data support the concept that IRE1 kinase activity rather than RNase activity regulates lipolysis induced by inflammatory, but not hormonal, stimuli, independent of canonical UPR signaling in adipocytes.

**Figure 3:**
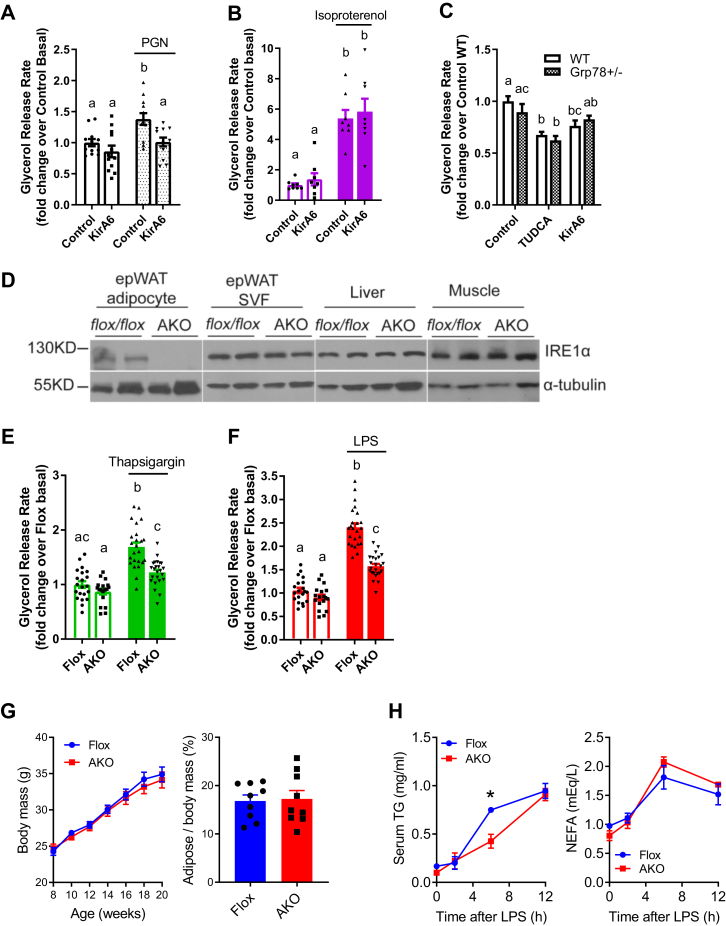
**IRE1 mediates lipolysis induced by inflammatory ligands in adipose tissue.***A* and *B*, glycerol release rates were determined in adipose tissue explants from wild-type C57Bl/6J mice treated with or without PGN (10 μg/ml) or isoproterenol (2 μM) in the absence or presence of the IRE1 inhibitor KirA6 (1 μM). Glycerol release rates were expressed relative to the control basal group (N = 8–12). *C*, adipose tissue explants were prepared from wild-type and Grp78 heterozygous C57Bl6/J littermate mice and treated with vehicle, TUDCA (1 mg/ml), or KirA6 (1 μM) for 72 h. Glycerol release rates were determined and expressed relative to the wild-type control group (N = 12). *D*, representative immunoblots of IRE1 from adipocytes and SVF of epididymal adipose tissue, as well as from the liver and muscle from littermate control (Flox) mice and adipocyte-specific IRE1 knockout (AKO) mice. *E* and *F*, glycerol release rates were measured in epididymal adipose tissue explants from flox littermate control versus AKO mice. Explants were treated with or without 1 μM thapsigargin (*E*) or 500 ng/ml LPS (*F*) for 72 h, and glycerol release was measured and expressed relative to the flox basal group (N = 18–24 explants). *G*, body mass in flox littermate control versus AKO mice aged 8–20 weeks (N = 13) and adipose tissue mass/body mass in 20-week-old mice (N = 9). *H*, serum triglycerides (TG) and nonesterified fatty acids (NEFA) from flox littermate control mice versus AKO mice before (*i.e.* 0 h), 1 h, 6 h, and 12 h after LPS injection (*i.p*. 1 mg/kg) (N = 4).Values are mean ± SEM. Statistical significance was measured as *p* < 0.05 using two-way ANOVA. Post hoc analysis was performed using Tukey’s multiple comparisons test. Conditions with different letters (a, b) denote a statistical difference compared with all other conditions without the same letter. ∗ denotes a statistical differences between flox and AKO mice

We next measured glycerol release in mouse-derived epididymal adipose tissue explants. Adipose tissue from wild-type (WT) C57Bl6/J mice showed that PGN (10 μg/ml) and isoproterenol (2 μM) increased glycerol release, but only the increased lipolysis caused by PGN was inhibited by KirA6 (Fig. 3, *A* and *B*). KirA6 did not alter isoproterenol-stimulated lipolysis in adipose tissue (Fig. 3*B*). We then tested if the canonical UPR was involved in ER stress or IRE1-related lipolysis using Grp78^+/-^ and WT littermate mice. We found that neither TUDCA nor KirA6 altered glycerol release in adipose tissue explants derived from Grp78^+/-^ mice, which had comparable lipolysis to WT mice (Fig. 3*C*). These data support our observations in 3T3-L1 adipocytes that IRE1 regulates lipolysis induced by inflammation independent of canonical UPR signaling and that IRE1 does not alter lipolysis from adrenergic stimuli in adipose tissue. Finally, we generated mice with adipocyte-specific knockout of IRE1 (*Ern1* gene), where protein levels of IRE1 were lower/absent in adipocytes from epididymal white adipose tissue (epiWAT), but not in the stromal vascular fraction (SVF), liver, or muscle from these mice (Fig. 3*D*). Adipose tissue explants from these IRE1 adipocyte-specific knockout mice (AKO) had lower glycerol release in response to thapsigargin or LPS when compared with floxed control littermate mice (Fig. 3, *E* and *F*). This data reinforces our findings using chemical inhibitors of IRE1 (*i.e.*, KirA6) in 3T3-L1 adipocytes and provides direct genetic evidence that IRE1 mediates lipolysis induced by inflammatory or bacterial ligands in mouse adipose tissue.

Finally, we tested the requirement for adipocyte IRE1 to regulate lipolysis after an inflammatory stress *in vivo*. We measured the time course of changes in serum lipids after LPS injection in AKO mice and floxed control littermate mice. Importantly, AKO mice and floxed control littermate mice have similar body mass and adipose mass (Fig. 3*G*). AKO mice and floxed control littermate had similar basal serum triglycerides and nonesterified fatty acids (NEFA) before LPS injection (Fig. 3*H*). We found that LPS injection increased the level of triglycerides in the blood in floxed control littermate mice (Fig. 3*H*). However, the LPS-induced increase in triglycerides was ∼50% lower 6 h after LPS injection in AKO mice (Fig. 3*H*). LPS injection increased the level of serum NEFA to similar levels in both AKO and floxed control littermate mice (Fig. 3*H*). These data show that adipocyte-specific IRE1 deletion did not change fat mass or blood lipids in the absence of inflammation, but adipocyte-specific IRE1 deletion impairs the increase in some blood lipids, such as triglycerides at specific times during an *in vivo* inflammatory stress.

### IRE1 kinase links inflammation to NF-κβ and cytokine secretion

**Figure 4:**
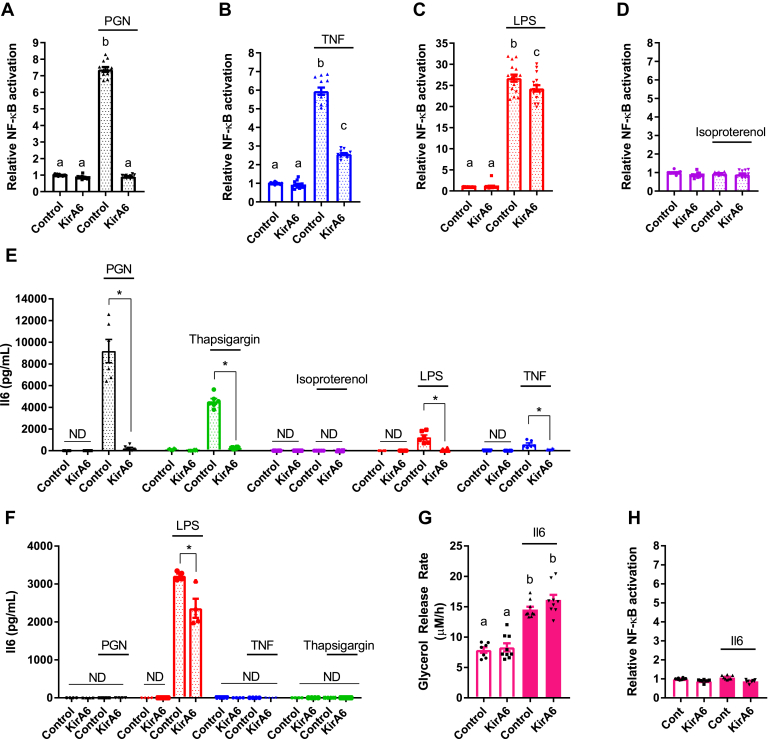
**Inflammatory ligands activate NF-kB and Il6 secretion through an IRE1 pathway in 3T3-L1 adipocytes.***A–D*, NF-κβ activation was measured in HEK-*Blue* NOD1 (*A, B, D*) or TLR4 (*C*) cells and reported relative to the vehicle control. Cells were treated with vehicle, 10 μg PGN (*A*), 10 ng/ml TNF (*B*), 500 ng/ml LPS (*C*), or 2 μM isoproterenol (*D*), in the presence or absence of 1 μM KirA6 (N = 12). *E*, Il6 secretion from differentiated 3T3-L1 adipocytes was measured by ELISA after 48 h of incubation in either the presence or absence of 1 μM KirA6, treated with vehicle, 10 μg/ml PGN, 1 μM thapsigargin, 2 μM isoproterenol, 500 ng/ml LPS, or 10 ng/ml TNF (N = 6). *F*, Il6 secretion from bone-marrow-derived macrophages was measured by ELISA after 48 h of incubation with or without 1 μM KirA6 and 10 μg/ml PGN, 500 ng/ml LPS, 10 ng/ml TNF, or 1 μM thapsigargin (N = 4). *G*, glycerol release rates were measured in 3T3-L1 adipocytes treated with or without Il6 (20 ng/ml) in the absence or presence of 1 μM KirA6 (N = 9). *H*, NF-κβ activation was measured *via* in HEK-*Blue* NOD1 cells with or without Il6 (20 ng/ml) in the absence or presence of 1 μM KirA6 (N = 8). Values are mean ± SEM. Statistical significance was measured as *p* < 0.05 using two-way ANOVA (*A–D*; *G*, *H*). Post hoc analysis was performed using Tukey’s multiple comparisons test. Conditions with different letters (a, b) denote a statistical difference compared with all other conditions without the same letter (*A–D*). Student *t-*test was used to determine statistical significance in *panels E* and *F*. ∗ denotes significance *p* < 0.05.

Activation of NF-κβ is a hallmark of inflammation, and we have previously reported that bacterial cell wall components (such as PGN and other NOD1 ligands) engage NF-κβ to stimulate adipocyte lipolysis (18). However, it is unknown if IRE1 acts upstream of NF-κβ to potentiate further cytokine secretion to augment lipolysis. HEK293 cells, stably expressing NOD1 or TLR4 with an NF-κβ SEAP reporter, were used to assess the involvement of IRE1 in activating NF-κβ in response to inflammatory stimuli. Treatment of HEK-NOD1 cells with either PGN (10 μg/ml FK565) or TNF (10 ng/ml), or HEK-TLR4 cells with 500 ng/ml LPS, increased NF-κβ activity, whereas isoproterenol (in HEK-NOD1 cells) did not activate NF-κβ (Fig. 4, *A–D*). KirA6 completely blocked NF-κβ activation induced by PGN and partially blocked NF-κβ activation induced by TNF. KirA6 had a very small, but statistically significant, inhibitory effect on LPS-induced NF-κβ activation (Fig. 4*C*). These data suggest that IRE1 kinase propagates inflammatory ligand-induced NF-κβ activation from NOD1 and TNFR, but biological relevance to TLR4 is not yet clear. Nevertheless, our data positions IRE1 kinase activity upstream of NF-κβ activation in responses to inflammatory triggers of lipolysis in adipocytes.

We next investigated if IRE1 kinase activity mediates inflammatory stimuli-induced cytokine secretion in adipocytes. Previous work has placed IRE1 upstream of NF-κβ in regulating Il6 secretion in macrophages (38). We found that 3T3-L1 adipocytes secrete Il6 in response to the inflammatory triggers PGN, LPS, TNF, and thapsigargin. KirA6 inhibited Il6 secretion stimulated by all inflammatory stimuli (PGN, LPS, TNF, thapsigargin), whereas isoproterenol did not stimulate Il6 secretion, in 3T3-L1 adipocytes (Fig. 4*E*). We also tested Il6 secretion in bone-marrow-derived macrophages (BMDMs). Only LPS stimulated Il6 secretion in BMDMs, which is consistent with previous results (40). LPS-stimulated Il6 secretion was partially inhibited by KirA6 in BMDMs (Fig. 4*F*). These data support a role for IRE1 kinase activity in mediating Il6 secretion in response to inflammatory stimuli in adipocytes.

Given the role of IRE1 in regulating Il6 secretion in response to inflammatory stimuli, we tested whether Il6 exposure induces IRE1 kinase-dependent lipolysis. Il6 increased glycerol release, but Il6-induced lipolysis was not altered by KirA6 (Fig. 4*G*). Furthermore, Il6 did not activate NF-κβ activity in HEK-293T cells stably expressing NOD1 and an NF-κβ SEAP reporter (Fig. 4*H*). Thus, our data is consistent with a model where IRE1 contributes to Il6 secretion from inflammatory triggers, but autocrine Il6 secretion does not feedback into an IRE1-dependent mechanism to stimulate lipolysis. Our data also show that not all cytokines engage IRE1 kinase to promote lipolysis, since KirA6 blocked TNF-stimulated, but not Il6-simulated, lipolysis in adipocytes.

### IRE1 kinase regulates lipolysis, independent of other tyrosine kinases

**Figure 5:**
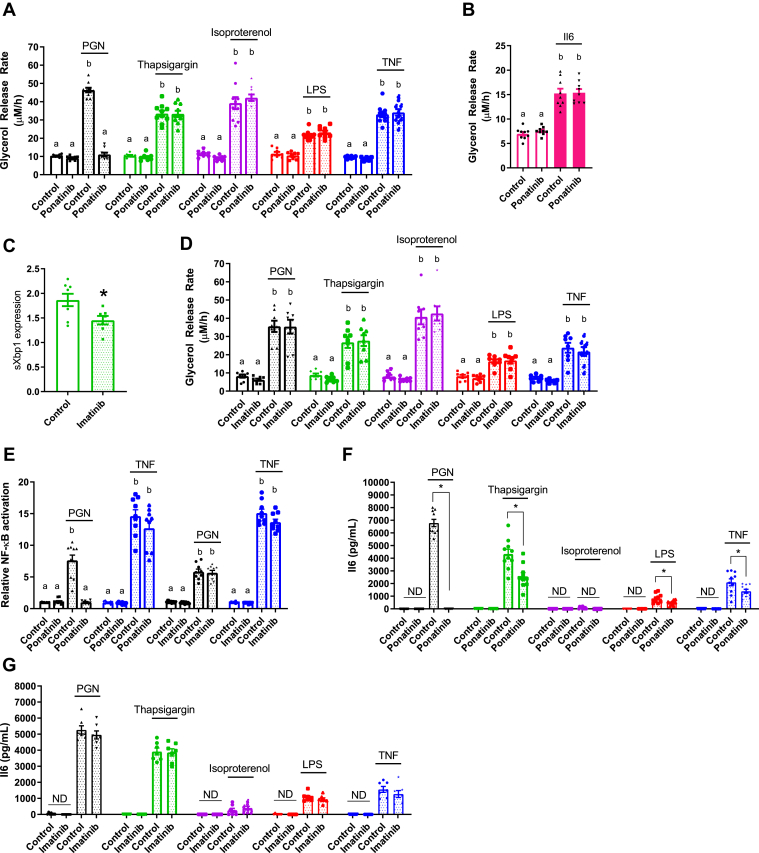
**The tyrosine kinase inhibitors ponatinib and imatinib do not alter the IRE1-mediated lipolysis in 3T3-L1 adipocytes.***A, B, D*, glycerol release rates were calculated in adipocytes treated with vehicle (control), 10 μg/ml PGN, 1 μM thapsigargin, 2 μM isoproterenol, 500 ng/ml LPS, 10 ng/ml TNF, or 20 ng/ml Il6, as indicated (N = 8–10). Cells were treated with ponatinib (0.1 μM) or imatinib (5 μM) for 1 h prior to ligand addition. *C*, Relative transcript levels of spliced XBP1 (sXBP1) in 3T3-L1 adipocytes treated with 0.25 μM Thapsigargin for 3 h with or without 5 μM imatinib. *E*, NF-κβ activation in HEK-*Blue* NOD1 cells treated with vehicle, 10 μg/ml PGN, or 10 ng/ml TNF in the presence or absence of 0.1 μM ponatinib or 5 μMimatinib (N = 8–9). *F* and *G*, Il6 secretion in 3T3-L1 adipocytes after 48 h of incubation with or without 0.1 μM ponatinib (*F*) or 5 μM imatinib (*G*) and 10 μg/ml PGN, 1 μM thapsigargin, 2 μM isoproterenol, 500 ng/ml LPS, or 10 ng/ml TNF, as indicated (N = 7–10). Values are mean ± SEM. Statistical significance was measured as *p* < 0.05 using two-way ANOVA (*A, B, D, E*). Post hoc analysis was performed using Tukey’s multiple comparisons test. Conditions with different letters (a, b) denote a statistical difference compared with all other conditions without the same letter. Student *t*-test was used to determine statistical significance in *panels C*, *F*, and *G*. ∗ denotes significance *p* < 0.05.

Multiple other kinases may mediate inflammation-induced lipolysis. For example, the ABL kinase, c-ABL, has been directly implicated in IRE1-linked ER stress responses (41). RIPK2 is a kinase required for NOD1-mediated NF-κβ activation and immune responses, and RIPK2 has also been reported to propagate ER stress and metabolic responses (38, 39, 42). We examined if these other kinase targets were involved in inflammation-induced lipolysis using the tyrosine kinase inhibitors (TKIs) ponatinib (0.1 μM) and imatinib (5 μM). Ponatinib has a high affinity for RIPK2, whereas imatinib potently inhibits c-ABL, but does not inhibit RIPK2 (43). Ponatinib completely blocked glycerol release stimulated by PGN, as expected due to its inhibition of RIPK2 (Fig. 5*A*). However, ponatinib failed to inhibit lipolysis stimulated by any other inflammatory or adrenergic stimulus (Fig. 5, *A* and *B*). Imatinib inhibited thapsigargin-induced sXBP1 levels in adipocytes (Fig. 5*C*), demonstrating that imatinib can inhibit downstream consequences of IRE1 RNase activity. However, imatinib did not affect increased lipolysis induced by any stimuli (Fig. 2*D*). These data indicate that RIPK2 is not required for all inflammation-stimulated lipolytic programs, rather RIPK2 is only involved in the NOD1-mediated lipolytic pathway. Furthermore, RIPK2 plays no role in lipolysis stimulated by adrenergic triggers of lipolysis such as isoproterenol. More importantly, the multiple targets inhibited by imatinib, such as ABL kinases, do not discriminate inflammatory versus hormonal-induced lipolysis. In fact, we find no role for the kinase targets of ponatinib and imatinib in regulating lipolysis beyond blocking RIPK2 responses to PGN. Seminal work showed that imatinib-mediated targeting of ABL-IRE1 alleviates ER stress in pancreatic beta cells *via* RNase activity and a UPR (41). We showed that imatinib can inhibit the target of IRE1 RNase activity, sXBP1, in thapsigargin-treated adipocytes, but the multiple kinases inhibited by imatinib (including c-ABL) did not alter inflammation or adrenergic lipolysis.

We next examined if these TKIs affect inflammation-induced NF-κβ/cytokine responses in a different way compared with lipolysis. As expected, RIPK2 inhibition with ponatinib completely blocked PGN-stimulated NF-κβ activation, but ponatinib did not alter TNF-stimulated NF-κβ activation (Fig. 5*E*). Imatinib did not alter NF-κβ activation induced by PGN or TNF (Fig. 5*E*). Ponatinib completely blocked NOD1-stimulated Il6 secretion and significantly lowered Il6 secretion stimulated by thapsigargin, LPS, and TNF (Fig. 5*F*). Imatinib did not affect Il6 secretion stimulated by any stimulus (Fig. 5*G*). Hence, our data show that bacterial, cytokine, and ER stress ligands trigger an intracellular kinase response that is different for lipolysis compared with Il6 secretion. Clearly, RIPK2 is required for PGN-NOD1-stimulated Il6 secretion and lipolysis, but our data also shows that the kinases targeted by ponatinib also contribute to the Il6 response for other inflammatory stimuli, at least at the dose of ponatinib used in adipocytes (0.1 μM). Overall, our data is consistent with a model where IRE1 kinase activity specifically regulates a lipolytic program in response to inflammatory stimuli, but other kinases are engaged to regulate immune response such as cytokine secretion.

### Inflammation *via* IRE1 kinase does not affect insulin signaling through Akt

**Figure 6:**
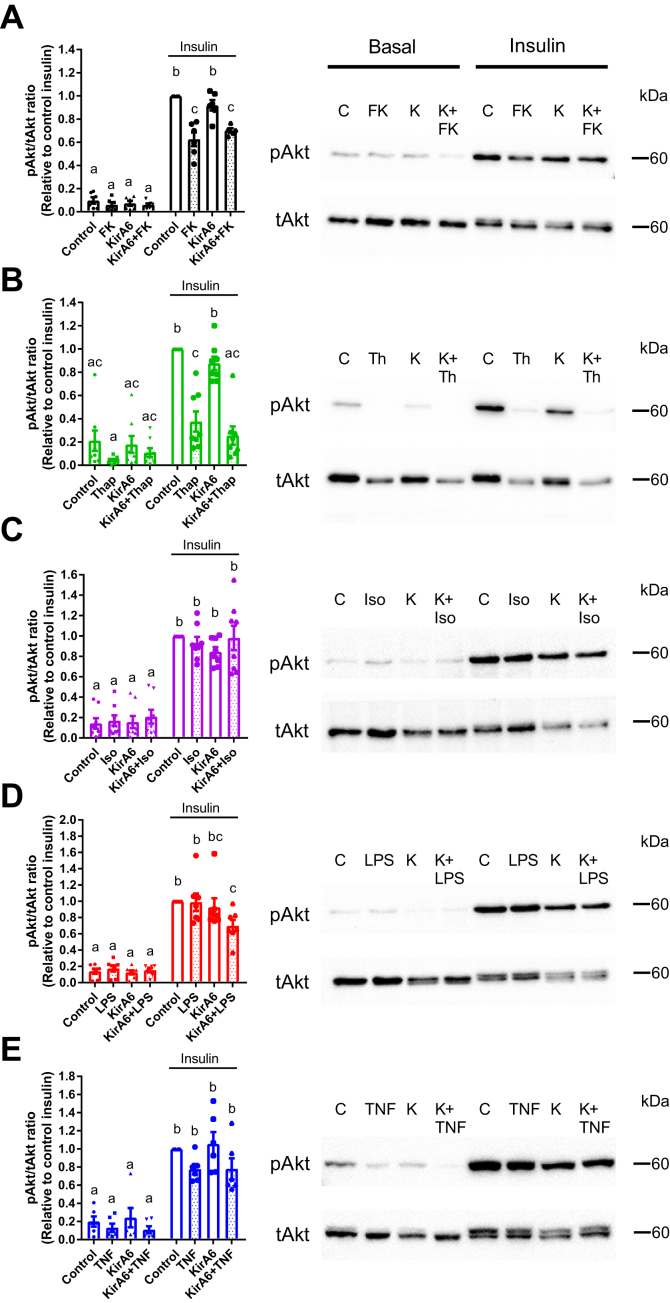
**IRE1 kinase activity does not affect insulin signaling through Akt.** Phosphorylation of Akt (pAkt) (Ser^473^) relative to total Akt (tAkt) was measured by immunoblotting in 3T3-L1 adipocytes in the absence or presence of 1 μM KirA6. Cells were pretreated with or without KirA6 for 1 h before addition of ligand for 48 h. Cells were treated with or without 100 nM insulin for the final 10 min after cells were exposed for 48 h to: *A*) PGN (10 μg/ml), *B*) thapsigargin (1 μM), *C*) isoproterenol (2 μM), *D*) LPS (500 ng/ml), or *E*) TNF (10 ng/ml). Control (C), FK565 (FK), KirA6 (K), thapsigargin (Th or Thap), isoproterenol (Iso), lipopolysaccharide (LPS), tumour necrosis factor (TNF). Values are mean ± SEM (N = 6–8). Statistical significance was measured as *p* < 0.05 using two-way ANOVA. Post hoc analysis was performed using Tukey’s multiple comparisons test. Conditions with different letters (a, b) denote a statistical difference compared with all other conditions without the same letter.

Inflammation and ER stress are known to promote insulin resistance (6, 8, 28). However, it is unknown if inflammatory triggers act through IRE1 kinase to promote adipocyte insulin resistance, which would then reduce the ability of insulin to suppress lipolysis. Exposure of adipocytes to PGN or thapsigargin inhibited phosphorylation of Akt in response to insulin (Fig. 6, *A* and *B*). However, inhibiting IRE1 kinase activity with KirA6 during PGN or thapsigargin exposure did not alter insulin signaling. In contrast, exposure of adipocytes to isoproterenol (Fig. 6*C*), LPS (Fig. 6*D*), or TNF (Fig. 6*E*) failed to inhibit insulin-stimulated phosphorylation of Akt. Thus, impaired insulin action is not a unifying factor underpinning adipocyte-autonomous lipolysis from inflammatory or adrenergic stimuli. Further, our data suggests that IRE1 kinase-dependent lipolysis in adipocytes can function independently of insulin resistance that can manifest from inflammation or ER stress.

## Discussion

**Figure 7:**
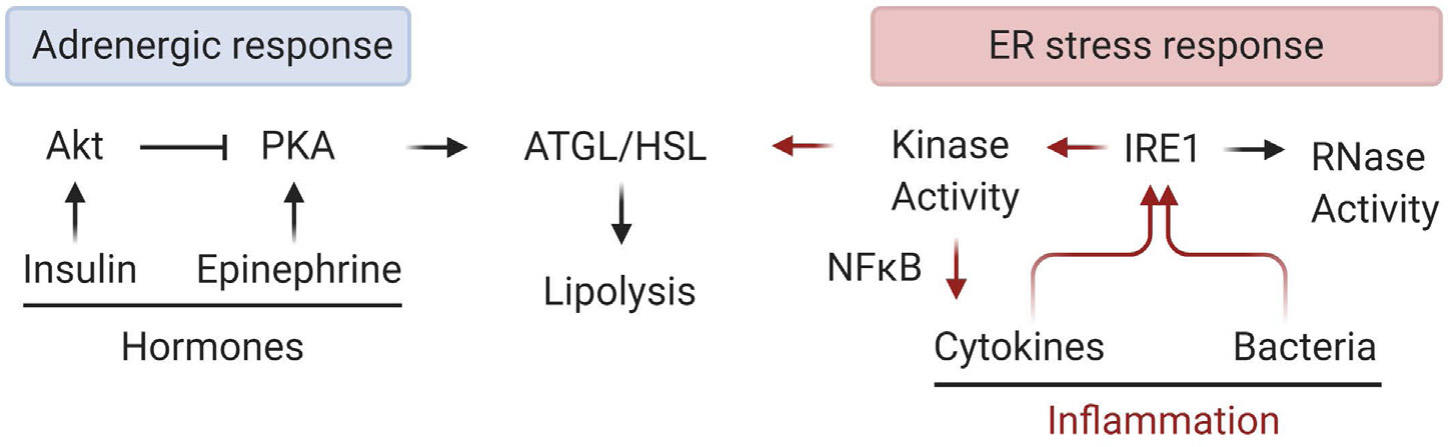
**IRE1 kinase activity promotes an inflammation-specific adipocyte lipolytic program that is separate from hormonal or adrenergic triggers of lipolysis.** Created with BioRender.com.

Obesity is associated with inflammation and increased adipocyte lipolysis. However, previous literature had never described a role for ER stress regulating a lipolytic response that is specific for inflammatory triggers of lipolysis (Fig. 7). We found that IRE1 kinase propagates inflammatory stress into increased lipolysis. Lipolysis can be increased through hormones and higher adrenergic activity or from inflammatory mediators inherent to adipose tissue during obesity. One connection between obesity-induced inflammation and hormonal control of lipolysis is insulin resistance, which manifests as impaired insulin-mediated suppression of lipolysis. Another key connection between inflammation and lipolysis showed that increased activation of ERK can stimulate Beta3 adrenergic receptor-mediated lipolysis *via* protein kinase A (PKA) (32). This outstanding discovery showed how a stress kinase could influence adrenergic-mediated lipolysis. Obesity is associated with increased ERK activity/phosphorylation preferentially in white adipose tissue (32). Obesity is also associated with higher insulin and increased levels of many potential triggers of inflammation within adipose tissue, including bacterial components and proinflammatory cytokines (5, 44). Indeed, cytokines and higher insulin can both activate ERK in adipose tissue, but it was not clear if triggers of inflammation engaged a different cellular response compared with hormones or if inflammation required insulin resistance to promote excessive lipolysis. It was also already known that ERK and PKA mediate part of the lipolytic response after adipocytes are exposed to bacterial components such as LPS or PGN (17, 18). However, it was not known if inflammatory mediators engage a specific kinase to promote a lipolytic program, which would represent a unifying factor for inflammation-induced lipolysis that is separate from hormonal triggers and adrenergic control of lipolysis.

ERK is a key regulator of HSL-mediated adipocyte lipolysis (45). However, ERK is not positioned to differentiate adrenergic and inflammatory lipolysis; rather ERK integrates cell stress and adrenergic signaling and represents a common node that promotes cross talk between stress and PKA/cAMP-mediated adipocyte lipolysis (32). Previous work has shown that inflammatory factors require different conditions to promote lipolysis compared with adrenergic stimuli. For example, TNF requires glucose in the media to stimulate adipocyte lipolysis, whereas extracellular glucose does not alter isoproterenol-induced lipolysis (46). Intriguingly, glucose did not alter the ability of TNF to stimulate proximal signaling events in lipolysis such as ERK activation. These data highlight the need to assess lipid/glycerol release (rather than just signal activation) and warrant scrutiny of the intracellular mechanism responsible for propagation of the lipolytic response due to inflammatory mediators. We assessed if a component of ER stress discriminated inflammatory triggers of lipolysis. The kinase activity of IRE1 was required for inflammatory triggers such as bacteria or certain cytokines to promote lipolysis.

Overall, our data support a model where the kinase activity of IRE1 is the component of ER stress that mediates inflammation-induced lipolysis (Fig. 7). Inflammatory stimuli require IRE1 kinase activity, but not RNase activity, to stimulate lipolysis. Adrenergic stimuli do not require IRE1 to promote lipolysis. Specific receptors for each inflammatory trigger converge on IRE1 to promote lipolysis. NOD1 activation requires RIPK2 to promote IRE1-mediated lipolysis and Il6 secretion. Not all cytokines take the same path to promote lipolysis, since TNF engages IRE1 and NF-κβ to increase lipolysis, but Il6 can circumvent these requirements.

Our results implicate IRE1 kinase activity in NF-κB and immune responses triggered by bacteria and certain cytokines, such as TNF. PKA can influence NF-κB, and we already knew that ERK, PKA, and NF-κB conspired to promote adipocyte lipolysis (18, 47). Our data show that IRE1 kinase activity is required for certain bacterial and cytokine triggers of immune responses to activate NF-κB. IRE1 kinase-mediated control of NF-κB is positioned to propagate lipolysis, but also a plethora of immune and stress responses related to NF-κB activation. It was important to define the role of IRE1-linked ER stress in regulating NF-κB and cytokines secretion because it was reported that the bacterial cell wall muropeptide sensors NOD1 and NOD2 are mediators of ER stress-induced cytokine secretion in macrophages (38, 39) and in the liver (48). It was shown that thapsigargin-induced ER stress promoted macrophage secretion of Il6, which was dependent upon IRE1, NOD1/2, and RIPK2 (38, 39). Our data show that the lipolytic and inflammatory pathways share dependence on IRE1, but do not share dependence on RIPK2. Our data show that RIPK2 does not function downstream of ER stress to promote Il6 secretion in adipocytes; rather, RIPK2 only mediates immune and lipolytic responses engaged by NOD1 or NOD2. Our results using a TKI that targets RIPK2 (*i.e.*, ponatinib) reinforce the concept that RIPK2 dictates immune, endocrine, and metabolic (*i.e.*, lipolysis) responses to PGN acting on NOD proteins (49). We also used the TKI imatinib to test the role of ABL kinases in ER stress-related lipolysis and immunity. This was important given the seminal work showing that inhibiting c-ABL with imatinib lowers IRE1-mediated RNase activity during the UPR (41).

Our data show no role for the UPR or RNase activity of IRE1 in the regulation of lipolysis triggered by inflammatory mediators. We found no correlation between changes in insulin signaling and lipolysis, and blocking IRE1 kinase activity had no effect on insulin signaling. Our data supports a model where bacterial and cytokine inflammatory stimuli promote lipolysis, NF-κB activation, and Il6 secretion through the kinase activity of IRE1. *In vivo* experiments showed that IRE1 within adipocytes was required for ∼50% of the increase in blood triglycerides caused by an inflammatory stimulus in mice. We found that adipocyte-specific deletion of IRE1 lowered serum triglyceride levels 6 h after an endotoxin challenge in mice. It is noteworthy that adipocyte-specific deletion of IRE1 did not alter serum NEFA responses after a bacterial endotoxin challenge. Adipocyte-specific IRE1 deletion also did not alter adipose mass or blood lipids in the absence of an inflammatory stress. Our data shows that IRE1 within adipocytes regulates lipolysis and blood triglycerides during inflammation, but IRE1 in multiple cell types could influence different aspects of lipid metabolism. There are many factors that can regulate the levels of specific blood lipids during inflammation and defining the role of IRE1 in adipocytes and other cells during integrated lipid metabolism responses during obesity and metabolic disease warrants further investigation. We propose that adipocyte resident IRE1 is a key regulator of lipolysis and that the distinction between the hormonal/adrenergic and inflammatory triggers of lipolysis is defined by IRE1 kinase (Fig. 7).

## Experimental procedures

### Materials

Heptanoyl-γ-D-glutamyl-L-meso-diamino-pimelyl-D-alanine (FK565) was obtained from Fujisawa Pharmaceuticals (Osaka, Japan). Fatty-acid-free bovine serum albumin (BSA; #A8806), thapsigargin (#T9033), isoproterenol (# I6504), Atglistatin (#SML1075), 3-isobytyl-1-methylanthine (#I5879), dexamethasone (#D4902), rosiglitazone (#R2408), insulin solution from bovine pancreas (insulin; #I0516), sodium tauroursodeoxycholate (TUDCA; #T0266), sodium phenylbutyrate (4-PBA; SML0309), and 8-Br-cAMP (#B7880) were from Sigma-Aldrich (St Louis, MO). Ponatinib (#A10080) and imatinib mesylate (imatinib; #A10468) were obtained from AdooQ Bioscience (Irvine, CA). Tumour necrosis factor (TNF; #575202) and Il6 (#575702) were from BioLegend (San Diego, CA). Dulbecco's Modification of Eagle's Medium (DMEM), Dulbecco’s phosphate-buffered saline (PBS), fetal bovine serum (FBS), and penicillin and streptomycin antibiotic solution (pen/strep) were obtained from Wisent (St Bruno, CA). GlutaMax, Restore Stripping Buffer, bicinchoninic acid (BCA) assay kit, SuperScript III (#18080085), and Amplitaq Gold (#4317742) were obtained from Thermo Fisher Scientific (Waltham, MA). Kinase inhibiting RNase Attenuator 6 (KirA6; #5322810001) and IRE1 inhibitor III 4μ8C (#412512) were obtained from Calbiochem. IRE1 RNase activity inhibitor KirA8 (#HY-114368) was obtained from MedChemExpress. Lipopolysaccharide (LPS; #tlrl-ppglps), Normocin (#ant-nr-1), Blasticidin (#ant-bl-1), Zeocin (#ant-zn-1), HEK-Blue Detection media (#hb-det3), HEK-Blue mNOD1 (#hkb-mnod1) cells, and HEK-Blue mTLR4 (#hkb-mtlr4) cells were obtained from InvivoGen (San Diego, CA). Antibodies for phospho-Akt (#4058) and total Akt (#9272) were obtained from Cell Signaling (Danvers, MA). Antibodies for phospho-S724-IRE1 (#ab48187) and total IRE1 (#ab37073) were from Abcam (Cambridge, MA).

### Differentiation and treatment of 3T3-L1 adipocytes

Murine 3T3-L1 preadipocytes were cultured in DMEM supplemented with 10% FBS, 1% GlutaMax, and 1% p/s (growth media) and maintained in an incubator at 37 °C and 5% CO_2_. Cells were seeded into 24-well plates in growth media and switched into differentiation media (growth media containing 0.5 mM 3-isobutyl-1-methylxanthine, 0.25 μM dexamethasone, 2 μM rosiglitazone, and 1 μg/ml insulin) 24 h postconfluence. After 48 h (Day 0–2), cells were incubated in growth media containing 1 μg/ml insulin for 96 h (day 3–6), with the media changed on Day 5. On Day 6, cells were placed in growth media and media was changed every 48 h until full differentiation was achieved. Experiments were performed between Days 8 and 10, depending on differentiation efficiency.

At the time of treatment, 3T3-L1 adipocytes were washed once with PBS and treated with or without indicated inhibitors for 1 h in serum-free DMEM containing 0.5% fatty-acid-free BSA and 1% p/s. Cells were treated with the general ER-stress inhibitors TUDCA or 4-PBA (varying concentrations), the Adipose Triglyceride Lipase (ATGL) inhibitor Atglistatin (5 or 50 μM), the IRE1 inhibitor KirA6 (1 μM, unless otherwise stated), the IRE1 RNase inhibitor KirA8 (10 μM, unless otherwise stated) or 4μ8C (30 or 60 μM) or the RIPK2 tyrosine-kinase inhibitor ponatinib (0.1 μM), or the tyrosine-kinase inhibitor imatinib (5 μM). After this 1-h incubation, one of the following ligands was added to the cells: PGN = 10 μg/ml FK565 (NOD1 activation), 500 ng/ml LPS (TLR4 activation), 10 ng/ml TNF, 20 ng/ml Il6, 1 μM thapsigargin (ER stress activation), 2 μM isoproterenol (β adrenergic activation), 0.5 mM 8-Br-cAMP, or a mock treatment.

### Glycerol release assay

Following treatments of 3T3-L1 adipocytes, supernatant samples were collected at 0, 24, and 48 h of ligand treatment (unless otherwise stated) and stored at –20 °C. Lipolysis was assessed *in vitro* by measuring the release of glycerol into the supernatant media over time (0, 24, 48 h) and calculating the glycerol release rate (μM/h). Glycerol concentration was measured as per the manufacturer’s protocol using the free glycerol determination kit (Sigma-Aldrich, #FG0100).

### Detection of IRE1 and Akt by western blotting

For determination of IRE1 phosphorylation, 3T3-L1 adipocytes were treated with or without the KirA6 inhibitor for 1 h and then the ligands FK565, thapsigargin, or isoproterenol were added for 3 h. Cells were lysed in lysis buffer containing 250 mM NaCl, 50 mM NaF, 5 mM EDTA, 10 mM Na_4_P_2_O_7_, 1 mM Na_3_VO_4_, 1% TX-100, 1 complete tablet of protease inhibitor, 50 mM Tris-HCl, and pH 7.4. Protein concentration was measured using a BCA assay kit (Sigma Aldrich) and spectrometry at absorbance 562 nm using a Synergy H4 Hybrid Reader (Biotek). Phospho-IRE1 was measured by western blot using 20 μg protein loading per sample. Sample polyvinylidene difluoride (PVDF) membranes were incubated in anti-phospho-S724-IRE1 (1:1000) antibody at 4 ˚C overnight and in goat anti-rabbit IgG horseradish peroxidase–conjugated secondary (1:5000) antibody for 1 h before detection using enhanced chemiluminescence detection (BioRad) and ChemiDoc Imaging (BioRad). PVDF membranes were stripped and reprobed for total IRE1 (1:1000). Phospho-IRE1 signal was quantified as the ratio between phospho/total IRE1 densitometry using ImageLab software (BioRad). Relative values were normalized to the control basal band of each membrane.

For determining phospho-Akt, 3T3-L1 adipocytes were treated with or without the KirA6 inhibitor for 1 h and then the ligands FK565, thapsigargin, isoproterenol, LPS, or TNF were added for 48 h. Cells were washed 1x with PBS and then treated with or without 100 nM insulin for 10 min before lysis. Protein concentration was detected as above, and phospho-Ser^473^-Akt (1:1000) was measured by western blotting using 20 μg protein loading per sample. PVDF membranes were stripped and reprobed for total Akt (1:1000). Phospho-Akt signal was quantified as the ratio between phospho/total Akt densitometry using ImageLab software, and relative values were normalized to the control insulin band of each membrane.

### Quantitative PCR (qPCR) detection of transcripts

For determining mRNA levels of select transcripts, 3T3-L1 adipocytes were treated with or without the KirA6 inhibitor for 1 h and then the ligands FK565, thapsigargin, or isoproterenol were added for 3 h. Cells were washed 1x in PBS before being suspended in Trizol. RNA was isolated, and cDNA synthesis was performed. Amplitaq Gold (Thermo Fisher Scientific) was used for qPCR using Taqman primers to BiP (Mm00517691), CHOP (Mm01135937), and sXbp1 (Mm03464496) in a Rotor-Gene-Q real-time PCR cycler (Qiagen). The gene Rplp0 (Mm01974474) was used as reference to calculate delta Ct values. Gene expression was expressed as fold change relative to basal control samples.

### Il6 ELISA

Media samples collected at 48 h of ligand treatment were analyzed for Il6 secretion. Il6 concentration was determined by using the Mouse Il6 DuoSet ELISA (R&D Systems, #DY406-05), as per the manufacturer’s protocol with the following modifications: the capture antibody was utilized at a working concentration of 1 μg/ml, and the detection antibody was 75 ng/ml.

### Determination of NF-κB activation

HEK-Blue NOD1 and HEK-Blue TLR4 cells stably express a secreted alkaline phosphatase (SEAP) reporter of NF-κB activity. HEK-Blue NOD1 cells were grown in media containing DMEM, 10% FBS, 1% GlutaMax, 1% p/s, 100 μg/ml Normocin, 30 μg/ml Blasticidin, and 10 μg/ml Zeocin (HEK-NOD1 media) and incubated at 37 °C and 5% CO_2_. HEK-Blue TLR4 cells were grown in media containing DMEM, 10% FBS, 1% GlutaMax, 1% p/s, 100 μg/ml Normocin, and 1x HEK-Blue selection (HEK-TLR4 media). Cells were seeded at a density of 30,000 cells/well into a 96-well plate and incubated for 24 h. Postincubation, cells were exposed to HEK-Blue detection media supplemented with 1 μM KirA6, 0.1 μM ponatinib or 5 μM imatinib, as appropriate. Cultures were allowed to equilibrate for 1 h before being treated with 10 μg/ml FK565, 500 ng/ml LPS, 10 ng/ml TNF, 20 ng/ml Il6, 1 μM thapsigargin, 2 μM isoproterenol, or a mock treatment. After 24 h, SEAP activity was measured by absorbance at 630 nm using the Synergy H4, Hybrid Reader (BioTek).

Adipose tissue explants and *in vivo* experiments in adipose-specific IRE1 knockout mice

Male C57Bl6/J mice aged 10–12 weeks were used for adipose tissue explant experiments unless otherwise indicated. These animal experiments were approved by McMaster University Animal Ethics Review Board. For experiments using Grp78^+/-^ mice, the WT controls were paired littermates. Gonadal fat pads were excised, cut into pieces ∼3–4 mg in size, and placed in DMEM containing 10% FBS and 1% pen/strep for 1 h at 37 ˚C and 5% CO_2_. Explanted tissues were then transferred to a second dish of DMEM containing 1% BSA and 1% pen/strep for 30 min. Next, 3–4 pieces of adipose tissue explant were placed per well into 24-well plates and treated with or without inhibitor (KirA6 or TUDCA) for 1 h. After 1 h, ligand was added to stimulate lipolysis and supernatant was collected at 0, 24, 48, and 72 h (inflammatory ligands) or at 0, 10, 30, 60 min (isoproterenol) for assessment of glycerol release. Explanted tissue was collected, dried, and weighed for normalization of the glycerol release data.

Adipocyte-specific IRE1 knockout (AKO) mice on the C57BL/6J background were generated by crossing *Adiponectin-Cre* mice (50) with *Ern1*^*flox/flox*^ mice (51). Epididymal fat pads were isolated from 8-week-old male AKO and *Ern1*^*flox/flox*^ control mice, cut into 3–4 mg pieces, and then placed in DMEM (high glucose) containing 10% FBS and 1% pen/strep for 1 h at 37 ˚C and 5% CO_2_. Fat explants were then transferred and incubated in DMEM containing 1% BSA (Sigma-Aldrich, A8806) and 1% pen/strep for 30 min before seeding into a 24-well plate with 3–4 pieces of explants per well in 1 ml DMEM (1% BSA and 1% pen/strep). After incubation for 1 h, Thapsigargin or LPS was added and the supernatants were collected at the indicated time for assessment of glycerol release. Explants were collected, dried, and weighed for normalization. Supernatant glycerol concentration was measured using the free glycerol determination kit (Sigma-Aldrich, F6428) and glycerol standard (Sigma-Aldrich, G7793). *In vivo* experiments were done by fasting mice for 6 h, followed by 1 h of refeeding (ad libitum), and collecting blood for lipid analysis; blood was collected at 0 h and then mice were injected with LPS (*i.p*. 1 mg/kg); and blood was collected 1, 6 and 12 h after LPS injection for serum lipid analysis. Blood triglycerides and NEFA were measured as described (18). These animal experiments were performed according to protocols approved by the Animal Care and Use Committee at the College of Life Sciences, Wuhan University.

### Bone-marrow-derived macrophage (BMDM) isolation and Il6 secretion

Bone marrow from the tibia and femur of C57BL6/J mice was harvested by flushing bones with DMEM and collecting the resultant cell suspension. Cells were centrifuged at 3500*g* at 4 °C for 5 min, and pelleted cells were washed in DMEM twice before plating in a 175 cm sq. flask for 3h in Macrophage Culture Media (MCM; DMEM +10%FBS +1% p/s + 15% L929-conditioned media). After 3 h, cells were gently washed, and adherent cells were discarded. Nonadherent cells were plated in 24-well plates at 2.5 x 10^5^ cells per well in MCM. L929-conditioned media was replenished on Days 3–4 (15% of total well volume), and cells were used for experiments between Day 7 and 10. Cells were treated with or without inhibitor (KirA6 or ponatinib) for 1 h before addition of inflammatory ligand (FK565, LPS, TNF, or thapsigargin). Media was collected after 48 h and Il6 secretion was tested by ELISA.

### Statistical analysis

Data are represented as the mean ± standard error of the mean (SEM). Comparisons were made using an unpaired, two-tailed Student’s *t*-test or two-way ANOVA and Tukey’s post hoc analysis, as appropriate using GraphPad Prism 7 software. During the study design, power calculations that were based on previous experience with the model system and methods informed sample size estimation. Cells and mice were randomly allocated into groups, all data were included and displayed, and results were confirmed from multiple passages of cells or tissues from multiple mice. Biological replicates are indicated and represent results from independent culture wells containing cells or tissues. Differences between groups were considered statistically significant when the *p*-value was less than 0.05.

## Data availability

All data described are located within this article, and all data are fully available upon request.

## Conflict of interest

The authors declare that they have no conflicts of interest with the contents of this article.
